# Exercise-Induced Muscle Damage and Recovery in Young and Middle-Aged Males with Different Resistance Training Experience

**DOI:** 10.3390/sports7060132

**Published:** 2019-05-29

**Authors:** John F. T. Fernandes, Kevin L. Lamb, Craig Twist

**Affiliations:** 1 Sport, Health and Well-being Arena, Hartpury University, Hartpury GL19 3BE, UK; 2 Department of Sport and Exercise Science, University of Chester, Chester CH1 4BJ, UK; k.lamb@chester.ac.uk (K.L.L.); c.twist@chester.ac.uk (C.T.)

**Keywords:** squatting, ageing, muscle damage

## Abstract

This study compared the time course of recovery after a squatting exercise in trained young (YG; *n* = 9; age 22.3 ± 1.7 years) and trained (MT; *n* = 9; 39.9 ± 6.2 years) and untrained (MU; *n* = 9; age 44.4 ± 6.3 years) middle-aged males. Before and at 24 and 72 h after 10 × 10 squats at 60% one-repetition maximum (1RM), participants provided measurements of perceived muscle soreness (VAS), creatine kinase (CK), maximal voluntary contraction (MVC), voluntary activation (VA), and resting doublet force of the knee extensors and squatting peak power at 20% and 80% 1RM. When compared to the YG males, the MT experienced *likely* and *very likely moderate* decrements in MVC, resting doublet force, and peak power at 20% and 80% 1RM accompanied by *unclear* differences in VAS, CK, and VA after the squatting exercise. MU males, compared to MT, experienced greater alterations in peak power at 20% and 80% 1RM and VAS. Alterations in CK, MVC, VA, and resting doublet force were *unclear* at all time-points between the middle-aged groups. Middle-aged males experienced greater symptoms of muscle damage and an impaired recovery profile than young resistance trained males. Moreover, regardless of resistance training experience, middle-aged males are subject to similar symptoms after muscle-damaging lower-body exercise.

## 1. Introduction

The number of middle-aged (i.e., 30 to 59 years old) people in the U.K. is increasing [1]. Alongside this is a growing number of middle-aged athletes, many of whom want to maintain or improve their athletic performances despite the natural age-related declines [2]. Specifically, these impairments are because of losses in muscle mass [3] and strength and power [3,4], of which, the lower-body undergoes the greatest losses [3,4,5]. Importantly, resistance training can provide a potent method of ameliorating these age-associated losses in muscle mass, strength, and power [6].

When used acutely, resistance exercise can cause exercise-induced muscle damage (EIMD; [6]), for which the mechanisms have been discussed extensively before (see [7]). EIMD symptoms include increases in muscle soreness, intramuscular enzymes in the blood serum, and plasma, and, of most importance to the athlete, an impaired muscle function [8]. Importantly, changes in muscle function provide the best indication of EIMD [7,8]. Although highly individualised [9], these symptoms typically peak between 24 and 48 h after the initial bout and are recovered by seven days [7]. A muscle’s susceptibility to damage might also be affected (reduced) in subsequent bouts where prior eccentric exercise has occurred [10,11]. Two studies have noted that this protection from eccentric exercise is less pronounced (~29% in MVC) in untrained older, compared to younger, men [12,13], which suggests that older resistance-trained men might exhibit symptoms of EIMD that are not dissimilar to their untrained counterparts.

Studies examining the recovery of older and younger untrained adults after muscle-damaging exercise are equivocal. Some studies have reported greater symptoms of EIMD in younger, compared to older, males [14,15], while others have observed greater EIMD in older (~59 to 66 years), compared to younger, males (~23 years) (17). Moreover, a number of studies have reported no difference in symptoms of EIMD after exercise for young populations (~19 years), compared to older populations (~48 to 76 years) [6,16,17,18,19]. One confounding factor in the current literature might be the physical activity and resistance training status of the participants. For example, when controlling for physical activity, Buford et al. [18] noted that recovery from muscle-damaging unilateral plantar flexion was similar among young (~23 years) and older (~76 years) adults. Despite the effectiveness of resistance training in combating the age-associated losses, only one study has investigated the EIMD response in older resistance trained males. Like Buford et al. [18], Gordon and colleagues [16] observed no differences in indirect markers of EIMD between recreationally trained young (~22 years) and middle-aged (~47 years) males after damaging knee extensor exercise. Despite these novel findings, no study has yet reported on the recovery characteristics from multi-jointed lower-body exercise in middle-aged (35 to 55 years), resistance trained males. Indeed, Gordon et al. [16] advised that future studies might adopt a more ecologically valid exercise protocol. Data from such a study would be highly applicable to those athletes seeking to prolong their athletic careers. Consequently, the primary aim of the study was to determine the time course to recovery from EIMD in young and middle-aged resistance trained males. A secondary purpose was to determine if the recovery profile of middle-aged males is altered by resistance training experience. Given the variability in the current data regarding EIMD and ageing and a lack of studies in trained populations, we propose the null hypothesis, i.e., that the EIMD response would not be different between groups.

## 2. Materials and Methods

### 2.1. Design

**Figure 1:**
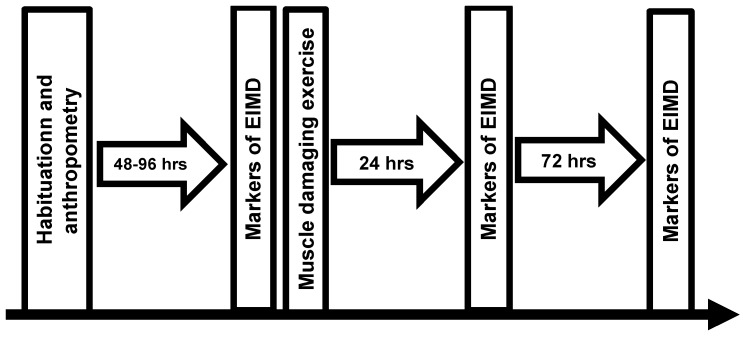
Schematic of study design.

The study used a two-way repeated measures design (age group x time), whereby participants attended the laboratory on four separate occasions, the initial visit for estimations of body composition and the back squat 1RM (Figure 1). On the same visit they were habituated with the measurements of squatting peak power and MVC, VA, and resting doublet force during isometric knee extension. Participants were considered ‘habituated’ when they could complete three consecutive repetitions that produced power or force values each within 10% [20]. Participants returned to the laboratory 2–4 days later for measurements comprising squats at 20% and 80% 1RM, MVC, VA, resting doublet force, muscle soreness, and creatine kinase (CK) activity, and an exercise bout comprising 10 × 10 squats at 60% 1RM [21]. Repeated measures were then conducted 24 and 72 h after the initial exercise bout.

### 2.2. Participants

Nine young resistance trained (YG; range: 21 to 25 years), nine middle-aged (MT; range: 35 to 54 years) resistance trained, and nine untrained middle-age males (MU; range: 35 to 53 years) were recruited for this study using convenience sampling. Thirty-five years was selected as the lower boundary for the middle-aged group because it is the entry age for ‘Masters’ athletes (see British Masters Athletic Federation and World Masters Athletics). As age-related studies typically use older groups (60 years and over), 55 was selected as the upper-limit for the middle-aged group. An overall sample size of approximately 27 (nine per group) was estimated using Batterham and Atkinson’s [22] nomogram. This was calculated using a coefficient of variation and typical change of 6.1% [23] and 5%, respectively. The YG and MT had a minimum of two years’ resistance training experience and regularly used squats as part of their resistance training programmes. The MU group had no resistance training experience, but was screened by the lead researcher to ensure they could perform the correct squat technique. All participants had been active in sport for a minimum of two years and were competitive. Participants completed a pre-test health questionnaire and provided written consent for the study, which was approved by the Ethics Committee of the Faculty of Life Sciences at the host institution. Participants were instructed not to consume any ergogenic supplements (for example, caffeine) on the day of testing and to refrain from exercise, other than that performed as part of the study, throughout their involvement.

### 2.3. Procedures

#### 2.3.1. Anthropometric Measurements

Body density was estimated via skinfold thickness measurements (Harpenden, British Indicators, Burgess Hill, UK) taken at the triceps, axilla, abdominal, suprailiac, chest, subscapular, and mid-thigh [24]. Body fat percentage (%BF) was estimated [25] from which quantities (kg) of fat-mass (FM) and fat-free mass (FFM) were derived.

#### 2.3.2. Resistance Training History and Sports Participation

The YG and MT participants completed a questionnaire to record how many years they had participated in regular resistance training, their weekly training frequency and session duration, and the main reason for their training. A second questionnaire detailed how many years they had participated in organised sport, their weekly frequency and session duration, and the type of sport they in which participated (i.e., team, endurance, racket, or other).

#### 2.3.3. Maximal Strength Testing

The 1RM for squat exercise was predicted using a three-repetition maximum (3RM) protocol. Participants performed 8–10 repetitions with 50% of their estimated 1RM, followed by 3–5 repetitions with 85% of their estimated 1RM. The load was then set at the approximate 3RM and the participants performed three repetitions. The load was progressively increased until the participant could no longer perform a complete repetition. The final load lifted was then used with the following equation [26] to estimate the 1RM squat load:

1RM = (100 × 3RM load lifted)/[48.8 + (53.8 × 2.71828^−0.075^ × repetitions). (1)

The above equation has been reported to yield accurate 1RM predictions (*r* = 0.969, 0.02% different from direct 1RM) [27].

#### 2.3.4. Indirect Markers of Muscle Damage

Perceived muscle soreness of the knee extensors was measured using a 0–10 visual analogue scale (VAS). Plasma CK activity was also determined from a capillary blood sample. A 30 µL sample of whole blood was collected into a heparinised capillary tube and pipetted onto a test strip for analysis (Reflotron, Type 4, Boehringer Mannheim, Mannheim, Germany).

#### 2.3.5. Assessment of Maximal Voluntary Contraction and Voluntary Activation

Before undertaking the MVC and VA assessments, participants performed a warm-up comprising five minutes of cycling at 100 W (Lode, Corival, Groningen, Netherlands). An isometric dynamometer (Biodex, Multi-joint system 3, Biodex Medical, New York, NY, USA) was employed to measure the force of the participant’s dominant knee extensor at 80° knee flexion. To prevent extraneous body movements, Velcro straps were applied tightly across the chest and thigh. Participants were provided with strong verbal encouragement and real-time feedback via the PC monitor.

The knee extensors were electrically stimulated (5 s with two 100 Hz single square impulses (doublet); Digitimer, D57, Hertfordshire, UK) using two 5 × 13 cm moistened surface electrodes (Axelgaard Manufacturing Co., Ltd., Fallbrook, CA, USA); one placed distally over the quadriceps and the other proximally over the upper quadricep. During optimisation, the amplitude of a doublet was progressively increased, starting at 50 amps, until a point where no further increases in intensity resulted in an increase in resting doublet force. Initially, a 230 volt electrically evoked doublet (set 20% above the value required to evoke a resting muscle doublet of maximum amplitude) was applied to the resting muscle (resting doublet) at 1 s. The resting doublet was used to elucidate any peripheral alterations that might have occurred as a result of the squatting protocol [21]. Participants then performed a 4 s MVC before a doublet, which was applied at the isometric plateau (superimposed doublet). The MVC was taken as the average force over 50 ms (AcqKnowledge 3 software, Biopac Systems, Massachusetts, MA, USA) before the superimposed doublet was applied. VA was calculated according to the interpolated doublet ratio using the equation:

VA (%) = [1 − (size of superimposed doublet/size of resting doublet)] × 100.(2)

A similar procedure has been deemed a reliable method (CV = 3.38%) for assessing VA [28].

#### 2.3.6. Assessment of Peak Power During Squat

Peak power was assessed at loads corresponding to 20% and 80% 1RM during the back squat exercise using a rotary encoder (FitroDyne, Fitronic, Bratislava, Slovakia), the procedures for which have been described elsewhere [5,23]. The FitroDyne has been shown to produce reliable intra- and inter-day measures of peak power (coefficient of variation = 3.9–6.1%) at the selected loads [23].

#### 2.3.7. Muscle-Damaging Exercise Protocol

This consisted of 10 × 10 repetitions of squat exercise at a load corresponding to 60% 1RM with 120 s rest between sets [21]. Each repetition was performed in the manner outlined above. A similar protocol has successfully induced symptoms of muscle damage in previous research [21,29]. The FitroDyne was used to calculate the power for each repetition in the manner outlined above. The average peak power per repetition was used to elucidate the influence of exercise intensity on recovery profiles between groups. One participant from the MU group was unable to complete sets 8, 9, and 10 at 60% 1RM, thus the load was reduced by 5 kg (50.1% 1RM) and power values were calculated accordingly.

### 2.4. Statistical Analyses

Comparisons of categorical training history and sport participation variables by group were made using a chi-squared (χ^2^) test of association. All other data were analysed using the effect size (ES) with 90% confidence intervals (CI) [30]. Magnitude-based inference statistics were used to provide information on the size of the differences, allowing for a more practical and meaningful explanation of the data [31]. Thresholds for the magnitude of the observed change for each variable were determined as the within-participant standard deviation in that variable × 0.2, 0.6, 1.2, and 2 for a small, moderate, large, and very large effect [32]. Threshold probabilities for a meaningful effect, based on the 90% confidence limits (CL) were as follows: Less than 0.5% *most unlikely*, 0.5–5% *very unlikely*, 5–25% *unlikely*, 25–75% *possibly*, 75–95% *likely*, 95–99.5% *very likely*, and >99.5% *most likely*. Effects with confidence limits across a likely small positive or negative change were classified as *unclear* [30]. All calculations were completed using predesigned spreadsheets (www.sportsci.org). Data are presented as ES, lower CI, and upper CI.

## 3. Results

### 3.1. Biometric Measures and Training History

Age and sum of skinfolds were *most likely* and *likely* higher, respectively, in the MT groups compared to the YG group (Table 1). Differences in FM and body fat percentage between the YG and MT groups were *very likely*, while mass and squat 1RM were *unclear*. Age and FFM differences between the MT and MU groups were *likely moderate*, whilst all other biometric characteristics demonstrated *unclear* differences.

The MT group had *most likely* regularly resistance trained for longer than the YG (ES 2.29, CI 1.46, 3.13; Table 2), though their training was associated with a lower weekly frequency (χ*^2^* = 32.5, *p* < 0.05) and shorter session duration (χ*^2^* = 36.4, *p* < 0.05). Moreover, the MT group typically chose resistance training for strength and fat loss, whereas the YG trained for strength (χ*^2^* = 31.8, *p* < 0.05).

There were *very likely large* and *moderate* differences in sports participation for the MT compared to the YG and MU, respectively, with MT having more years compared to the YG (ES 1.47, CI 0.66, 2.28) and less than the MU group (ES 1.17, CI 0.36, 1.98; Table 3). No relationship (*p* > 0.05) was observed between groups for weekly frequency, session duration, or type of sport played.

### 3.2. External Load Response during the Muscle-Damaging Protocol

There was a *likely moderate* lower average peak power (ES −0.71 CI −1.53, 0.10) in the MT (603.2 ± 162.6 W) compared to the YG (770.4 ± 278.0 W). Differences between the MT and MU (547.0 ± 75.0 W) groups were *unclear* (ES −0.43, CI −1.25, 0.39).

### 3.3. Indirect Markers of Muscle Damage

**Figure 2:**
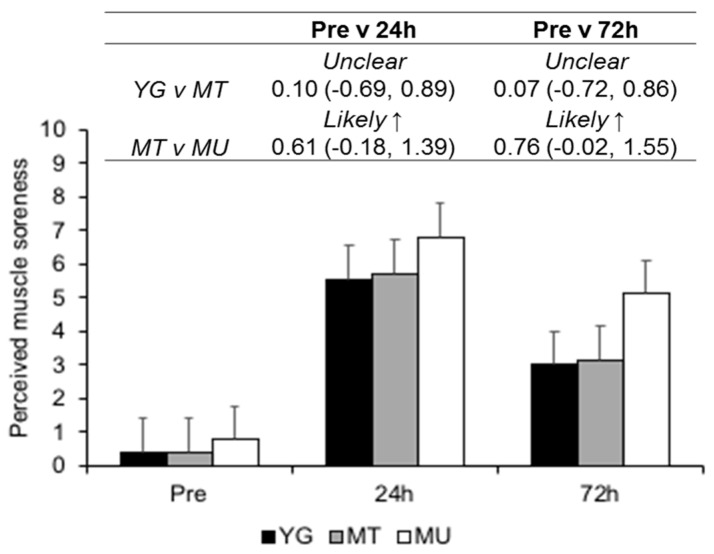
Changes in perceived muscle soreness between YG, MT, and MU at pre, 24, and 72 h after resistance exercise. The panel above details the qualitative descriptor, effect size, and upper and lower confidence limits.

At Pre, differences in muscle soreness between the YG and MT and MT and MU were *unclear* (ES 0.00, CI −0.81, 0.81 and ES 0.42, CI −0.39, 1.22, respectively; Figure 2). When the three groups were combined, perceived muscle soreness demonstrated *most likely very large* (ES 4.20, CI 3.74, 4.65) increases at 24 h and, likewise (ES 1.82, CI 1.36, 2.27), at 72 h after muscle-damaging exercise. Between-group differences for the YG and MT comparison were *unclear* at 24 and 72 h after muscle-damaging exercise. Increases in muscle soreness were *likely moderately* higher in the MU group compared to the MT group at 24 and 72 h.

**Figure 3:**
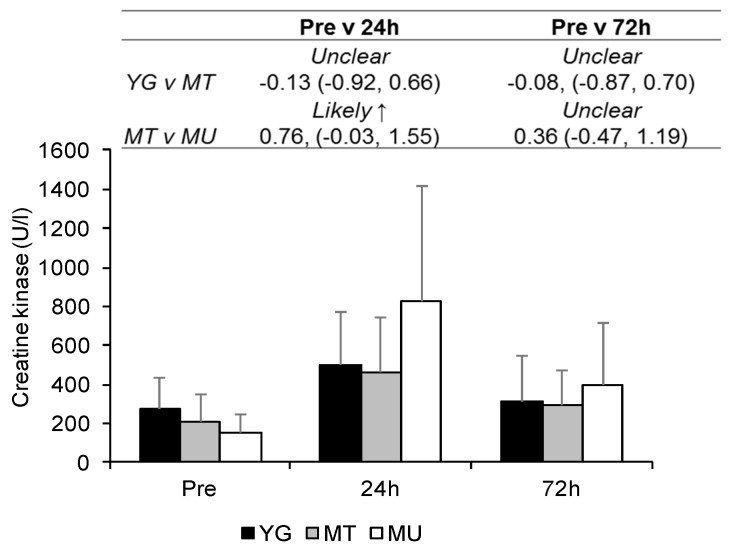
Changes in plasma creatine kinase activity between YG, MT, and MU at Pre, 24, and 72 h after resistance exercise. The panel above details the qualitative descriptor, effect size, and upper and lower confidence limits.

Differences in CK activity at Pre for YG and MT and MT and MU comparisons were *unclear* (ES −0.41, CI −1.21, 0.40 and ES −0.44, CI −1.25, 0.38, respectively; Figure 3). The increase in plasma CK activity for the three groups combined was *very likely moderate* (ES 1.19, CI 0.73, 1.64) and *likely small* (ES 0.59, CI 0.13, 1.05) at 24 and 72 h, respectively, compared to Pre. Differences in plasma CK activity over time were *unclear* between the YG and MT groups. Plasma CK activity was *likely moderately* higher in the MU group compared to the MT group at 24 h, though differences between the groups were *unclear* at 72 h.

**Figure 4:**
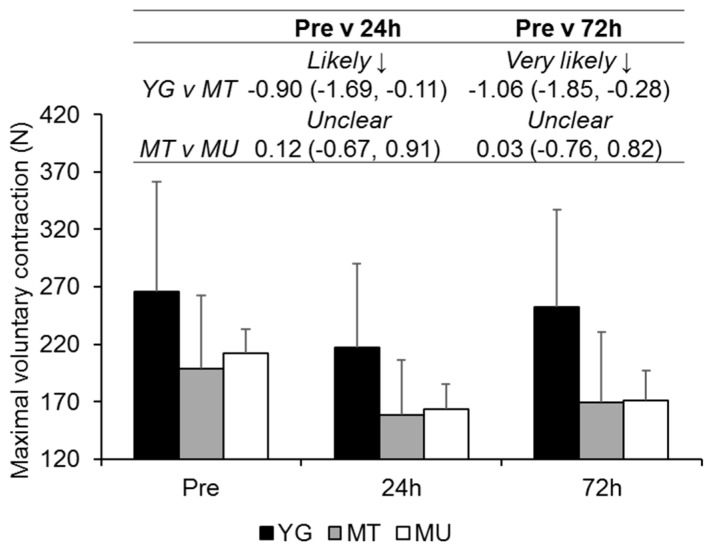
Changes in maximal voluntary contraction force between YG, MT, and MU at Pre, 0, 24, and 72 h after resistance exercise. The panel above details the qualitative descriptor, effect size, and upper and lower confidence limits.

At Pre, differences in MVC force were *likely moderate* and *unclear* for the YG compared to MT (ES −0.80, CI −1.61, 0.01) and MT compared to MU (ES 0.27, CI −0.56, 1.10), respectively (Figure 4). MVC force had *very likely moderate* (ES −0.71, CI −1.16, −0.26) and *likely small* (ES −0.39, CI −0.84, 0.06) decreases at 24 and 72 h after muscle-damaging exercise. *Likely* and *very likely moderate* reductions in MVC force were observed in the MT group compared to the YG groups at 24 and 72 h, respectively. At 24 and 72 h, differences between the MT and MU groups were *unclear.*

**Figure 5:**
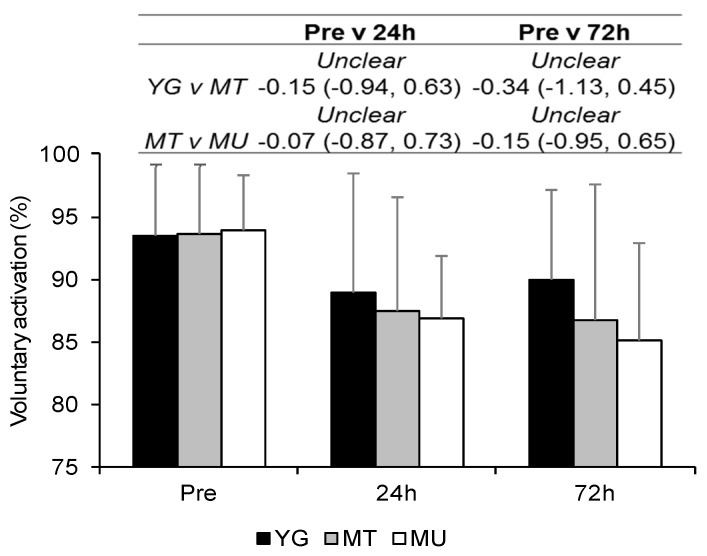
Changes in voluntary activation between YG, MT and MU at Pre, 24, and 72 h after resistance exercise. The panel above details the qualitative descriptor, effect size, and upper and lower confidence limits.

Differences in VA at Pre were *unclear* for YG compared to MT (ES 0.03, CI −0.77, 0.84) and MT compared to MU (ES 0.07, CI −0.76, 0.90; Figure 5). When all groups were combined VA decreased over time, with values at 24 and 72 h demonstrating *very likely moderate* decreases (ES −0.87, CI −1.33, −0.41 and ES −0.88, CI −1.34, −0.41, respectively). Differences between groups were *unclear* at all time-points.

**Figure 6:**
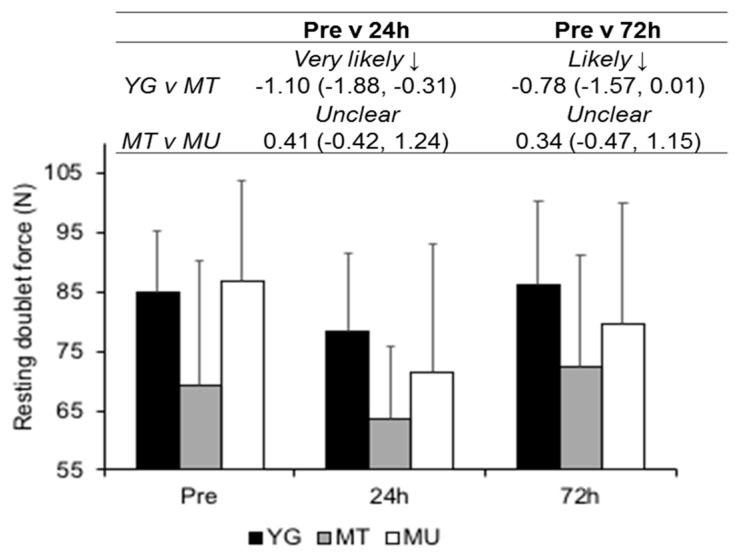
Changes in resting doublet force between YG, MT and MU at Pre, 24, and 72 h after resistance exercise. The panel above details the qualitative descriptor, effect size, and upper and lower confidence limits.

Higher mean resting doublet values for the YG were *likely moderate* compared to the MT (ES −0.96 CI −1.77, 0.14; Figure 6). Similarly, higher values for MU (ES 0.95, CI 0.12, 1.78) were *likely moderate* compared to the MT group. Mean doublet values were *likely small* and *unclear* at 24 and 72 h, respectively, (ES −0.52, CI −0.98, −0.06 and ES −0.04, CI −0.50, 0.42, respectively) after squatting exercise. Differences in resting doublet were *very likely moderate* and *likely moderate* between YG and MT groups at 24 and 72 h, respectively. MT and MU comparisons were *unclear* at 24 and 72 h.

### 3.4. Peak Power during Squat Exercise

*At Pre,* a *very likely moderate* lower peak power was at 20% and 80% 1RM (ES −1.03, CI −1.84, −0.22 and ES −1.03, CI −1.84, −0.21, respectively) was observed in the MT compared to YG (Table 4). Differences at Pre for MT and MU were *most likely very large* and *unclear* for 20% and 80% 1RM (ES −3.34, CI −4.18, −2.50 and ES −0.47, CI −1.28, 0.33, respectively). When all groups were combined, peak power for 20% and 80% 1RM demonstrated *possibly small* (ES −0.25, CI −0.71, 0.20 and ES −0.36, CI −0.81, 0.09, respectively) and *unclear* (ES −0.23, CI −0.69, 0.22 and ES −0.19, CI −0.64, 0.26, respectively) decrements at 24 and 72 h, respectively. For 20% and 80% 1RM, between group differences at 24 and 72 h were *very likely moderate* between the YG and MT groups. Similarly, reductions in 20% 1RM peak power at 24 and 72 h for the MT vs. MU comparison were *very likely moderate.* Peak power at 80% 1RM illustrated *likely moderate* and *very likely large* differences at 24 and 72 h, respectively.

## 4. Discussion

Contrary to our hypothesis, the current findings highlight the magnitude of exercise-induced muscle damage and time-course of recovery after lower body resistance exercise is greater in trained middle-aged males than their young counterparts. Moreover, regardless of resistance training experience, middle-aged males experienced like symptoms of muscle damage and a similar recovery profile in the days after.

### 4.1. Confirmation of EIMD

The small to moderate loss of force at 24 and 72 h observed in the current study confirms that the prescribed lower-body resistance exercise caused EIMD. Although not indicative of myofibrillar disruption [7,8], the small to very large increases in muscle soreness and CK activity indicate that tissue damage occurred after squatting exercise. The losses in MVC support previous observations of isometric strength loss after lower-body eccentric exercise in younger resistance trained males [21]. The reductions in MVC at 24 h possibly owe to both peripheral and central impairments, given the contemporaneous decrements in resting doublet and VA. However, that resting doublet scores were recovered by 72 h, but VA remained suppressed, suggests that the reductions in MVC at the later time point were caused by central alterations. Potential central mechanisms include a reduction in drive to the muscle caused by neural impairments and reduction in excitability to the alpha motor-neuron [28,33].

### 4.2. Changes in Indirect Markers of EIMD in Trained Young and Middle-Aged Males

That differences between trained groups on plasma CK activity after resistance exercise were unclear reaffirms the findings of previous studies [15,18,34], suggesting that membrane permeability is similar between trained young and middle age groups. Likewise, the comparable changes in muscle soreness observed in the two resistance trained groups is consistent with the work of Buford et al. [18], albeit in a non-resistance trained sample, in the plantar flexors, though contradictory to reports of greater soreness experienced by younger males after muscle-damaging elbow flexor exercise [14,19]. Increases in muscle soreness might reflect damage to connective tissue and decreases in range of motion, rather than damage to the contractile machinery *per se* [7,8]. Consequently, these data indicate that CK and muscle soreness responses to lower-limb muscle damaging exercise are similar in young and middle-aged resistance trained males.

### 4.3. Changes in Muscle Function in Trained Young and Middle-Aged Males

Reductions in MVC, VA, and resting doublet occurred in both resistance trained groups after EIMD. The finding that Pre VA values were not different between groups contrasts previous suggestions that older healthy adults are unable to activate the muscle to the same extent as their young counterparts [35], possibly owing to the trained nature of the MT group [36]. That the time course of VA recovery after high volume squatting exercise was not different between the MT and YG groups is also a novel finding. The moderately greater reductions in MVC in the MT group, compared to the YG group after EIMD, appear to be mediated by peripheral alterations (i.e., disruptions of sarcomeres and impaired excitation-contraction coupling), as reflected by the lower resting doublet values in the older trained participants. Given that differences in VA were unclear between the resistance trained groups after EIMD suggests that central alterations are not responsible for the greater reductions in MVC in the MT group.

The lower Pre peak power values at 20% and 80% 1RM in the MT group, compared to the YG group, are similar to those previously reported in resistance trained middle-aged males [5]. For the first time, this study has highlighted that the decrements in peak power after EIMD are of a greater magnitude in middle-aged males, compared to young resistance trained males. Work in young athletes indicates that lower-body power output has strong relationships with a variety of sporting tasks [37,38]. Thus, it is plausible that the impaired power output due to EIMD may inhibit these movements in trained young and middle-aged males. Applied practitioners should therefore be cognisant of this and consider adopting different recovery practices for young and middle-aged male athletes after muscle-damaging lower-limb exercise.

### 4.4. Differences in Recovery Between Trained and Untrained Middle-Aged Males

The two middle-aged groups produced similar peak power during the muscle-damaging protocol, which was followed by similar changes in MVC, VA, resting doublet, and CK. The repeated bout effect (RBE) [7,10] suggests that resistance trained males should experience less muscle damage after eccentric exercise compared to untrained males. However, the attenuated protection offered to the muscle with ageing [12,13] might explain the similar recovery profiles in these age groups. Moreover, the similar sporting characteristics of the two middle-aged groups might also explain why both demonstrated a comparable recovery profile. That is, the training experienced by both groups during their sports participation might have provided a similar protection to the muscle-damaging squatting exercise. A further explanation might be owed to the similar peak power produced during the muscle-damaging protocol. It has been noted previously that the magnitude of EIMD and recovery were positively related to the workload during the muscle damaging protocol in young and older adults [39]. Given that both middle-aged groups produced a similar peak power during the exercise protocol, it is perhaps not unexpected that the recovery profile was similar. After high volume squatting, differences between middle-aged groups in perceived muscle soreness and peak power were moderate to large. After muscle damaging exercise, the MU group demonstrated greater losses in peak power compared to the MT group. It is plausible that the resistance training experience of the MT group served to preserve or enhance the type 2 fiber cross-sectional area [40], thus accounting for their smaller losses in peak power. Consequently, resistance training in middle-aged males might help to maintain lower-body peak power after muscle-damaging exercise, but does not appear to alter other indirect markers of EIMD.

### 4.5. Limitations

Readers should be aware of the cross-sectional nature of this study. That is, cause and effect cannot directly be established, but rather, only associations between age groups and different training status. However, given the large differences between age groups (>18 years), designing a study that spanned over ~18 years would be unfeasible. Whilst the high variability in plasma CK in our sample is concerning, it should be noted that CK alterations show a poor temporal pattern with muscle function [41]. As such, the CK alterations should be used to confirm tissue damage, rather than indicate the magnitude of muscle damage.

## 5. Conclusions

This study reports that the magnitude of EIMD, as indicated by a reduction in muscle function, and time-course of recovery after high volume resistance exercise is greater in trained middle-aged males compared to their young counterparts. Practically, trained middle-aged males should be cognisant of requiring greater recovery time and adopt appropriate strategies. Moreover, resistance training in middle-aged males could attenuate the losses in peak power after high volume squatting exercise, but does not alter the recovery profile of other indirect markers of muscle damage. Applied practitioners should be mindful of these alterations in trained and untrained middle-aged males and should programme training accordingly.

## Figures and Tables

**Table 1:** Biometric characteristics (mean ± SD) and comparisons of young (YG) and middle-aged trained (MT) and untrained (MU) groups.

Measure	Group			Comparison	
	YG (*n* = 9)	MT (*n* = 9)	MU (*n* = 9)	YG v MT	MT v MU
Age (years)	22.3 ± 1.7	39.9 ± 6.2	44.4 ± 6.3	*Most likely ↑* 3.70 (2.87, 4.53)	*Likely ↑* 0.71 (−0.10, 1.52)
Mass (kg)	82.0 ± 9.0	79.1 ± 10.3	83.4 ± 9.56	*Unclear* 0.29 (−1.10, 0.52)	*Unclear* 0.42 (−0.39, 1.23)
Fat-free mass (kg)	71.4 ± 7.9	63.9 ± 6.5	68.6 ± 7.1	*Very likely ↓* −1.02 (−1.83, −0.22)	*Likely ↑* 0.68 (−0.13, 1.49)
Fat-mass (kg)	10.5 ± 4.5	15.2 ± 5.7	14.8 ± 7.0	*Likely ↑* 0.89 (0.09, 1.70)	*Unclear* −0.07 (−0.88, 0.74)
Body fat (%)	12.8 ± 4.7	18.8 ± 5.8	17.4 ± 6.7	*Very likely ↑* 1.13 (0.32, 1.94)	*Unclear* −0.23 (−1.04, 0.58)
Sum of skinfolds (mm)	82.3 ± 24.6	102.4 ± 31.9	91.7 ± 32.7	Likely *↑* 0.69 (−0.12, 1.50)	*Unclear* −0.32 (−1.13, 0.48)
Squat 1RM (kg)	130.8 ± 26.8	109.3 ± 22.5	98.4 ± 14.25	*Unclear* −0.85 (−1.65, −0.04)	*Unclear* −0.56 (−1.37, 0.25)

The comparison panel details the qualitative descriptor, effect size, and upper and lower confidence limits.

**Table 2:** Resistance training characteristics of the young (YG) and middle-aged trained groups (MT).

Resistance Training Characteristics		YG (*n* = 9)	MT (*n* = 9)
Years of resistance training (mean ± SD)		4.6 ± 1.3	18.0 ± 5.6
Weekly frequency *	1 to 2	2 (22.2)	6 (66.7)
	3 to 4	4 (44.4)	2 (22.2)
	5+	3 (33.3)	1 (11.1)
Session duration *	0 to 30 min	0 (0.0)	1 (11.1)
	31 to 60 min	3 (33.3)	7 (77.8)
	61 to 90 min	5 (55.6)	1 (11.1)
	90+ min	1 (11.1)	0 (0.0)
Reason for resistance training *	Strength	6 (66.7)	4 (44.4)
	Hypertrophy	1 (11.1)	0 (0.0)
	Fat loss	1 (11.1)	4 (44.4)
	Health	1 (11.1)	1 (11.1)

* Categorical variables are significantly associated (*p* < 0.05). Brackets denote percentage of responses in each category.

**Table 3:** Sports participation characteristics of the young and middle-aged trained groups.

Sports Participation Characteristics		YG (*n* = 9)	MT (*n* = 9)	MU (*n* = 9)
Years of sports participation (mean ± SD)		11.2 ± 4.8	22.0 ± 7.8	30.3 ± 7.8
Weekly frequency	1 to 2	4 (44.4)	2 (22.2)	0 (0.0)
	3 to 4	4 (44.4)	4 (44.4)	6 (66.7)
	5+	1 (11.1)	3 (33.3)	3 (33.3)
Session duration	0 to 30 min	0 (0.0)	0 (0.0)	0 (0.0)
	31 to 60 min	3 (33.3)	4 (44.4)	7 (77.8)
	61 to 90 min	3 (33.3)	3 (33.3)	1 (11.1)
	90+ min	3 (33.3)	2 (22.2)	1 (11.1)
Type of sport	Team	5 (55.6)	3 (33.3)	3 (33.3)
	Endurance	3 (33.3)	5 (55.6)	4 (44.4)
	Racket	0 (0.0)	1 (11.1)	2 (22.2)
	Other	1 (11.1)	0 (0.0)	0 (0.0)

**Table 4:** Peak power at Pre, 24 and 72 h.

Intensity	Group	Pre	24 h	72 h	Comparison (90% CI)	
					Pre v 24 h	Pre v 72 h
20% 1RM (W)	YG	507.9 ± 134.6	473.8 ± 119.9	476.6 ± 119.7	YG v MT	
					*Very likely ↓*	*Very likely ↓*
	MT	387.4 ± 87.9	360.3 ± 76.1	366.3 ± 76.4	−1.07(−1.85, −0.28)	−1.04 (−1.82, −0.25)
					MT v MU	
	MU	320.7 ± 47.9	291.7 ± 40.1	289.7 ± 40.2	Very likely *↓*	Very likely *↓*
					−1.06 (−1.84, −0.27)	−1.17 (−1.96, −0.39)
80% 1RM (W)	YG	1295.3 ± 369.1	1207.5 ± 328.2	1275.9 ± 338.3	YG v MT	
					Very likely *↓*	Very likely *↓*
	MT	977.1 ± 211.1	869.8 ± 195.0	964.9 ± 212.1	−1.07 (−1.96, −0.39)	−1.04 (−1.83, −0.25)
					MT v MU	
	MU	886.0 ± 163.2	746.7 ± 153.3	735.1 ± 134.8	Likely *↓*	Very likely *↓*
					−0.67 (−1.45, 0.12)	−1.22 (−2.01, −0.43)

The comparison panel details the qualitative descriptor, effect size, and upper and lower confidence limits.
